# Role of matrix metalloproteinases in diabetic foot ulcers: Potential therapeutic targets

**DOI:** 10.3389/fphar.2022.1050630

**Published:** 2022-10-20

**Authors:** Kang Fu, Xueyao Zheng, Yuhan Chen, Liuying Wu, Zhiming Yang, Xu Chen, Wei Song

**Affiliations:** ^1^ School of Life Sciences, Hubei University, Wuhan, China; ^2^ National & Local Joint Engineering Research Center of High-throughput Drug Screening Technology, State Key Laboratory of Biocatalysis and Enzyme Engineering, Hubei University, Wuhan, China

**Keywords:** diabetic foot ulcers, matrix metalloproteinases, tissue inhibitors of metalloproteinases, MMP inhibitors, wound healing

## Abstract

Diabetic foot ulcers (DFUs) are pathological states of tissue destruction of the foot or lower extremity in diabetic patients and are one of the serious chronic complications of diabetes mellitus. Matrix metalloproteinases (MMPs) serve crucial roles in both pathogenesis and wound healing. The primary functions of MMPs are degradation, which involves removing the disrupted extracellular matrix (ECM) during the inflammatory phase, facilitating angiogenesis and cell migration during the proliferation phase, and contracting and rebuilding the tissue during the remodeling phase. Overexpression of MMPs is a feature of DFUs. The upregulated MMPs in DFUs can cause excessive tissue degradation and impaired wound healing. Regulation of MMP levels in wounds could promote wound healing in DFUs. In this review, we talk about the roles of MMPs in DFUs and list potential methods to prevent MMPs from behaving in a manner detrimental to wound healing in DFUs.

## Introduction

Diabetic foot ulcers (DFUs) are one of the serious chronic complications of diabetes mellitus and also a substantial cause of morbidity and mortality in developed countries (Røikjer et al., 2022). The lifetime chance of a diabetic patient having a foot ulcer has been reported to range between 19% and 34%. Due to reduced blood supply to the lower extremities, DFUs are likely to be subject to necrosis, infection, and deep tissue involvement, which may result in amputation (Lipsky et al., 2012; Centers for Disease Control and Prevention, 2014; Hingorani et al., 2016). The rate of lower limb amputations due to DFUs is 10–20 times higher than those without diabetes. The treatment of DFUs can be complicated and expensive. Healing rates with conventional treatments for DFUs have been found to range from 12% to 20% on average in clinical trials. Current managements of DFUs include offloading the wound, using dressings to maintain a moist wound environment, debridement when necessary, controlling infection and blood glucose(Everett and Mathioudakis, 2018). However, ulcer recurrence rates remain high: 40% within one year and 65% within five years after wound healing (Armstrong et al., 2017; Fu et al., 2019).

Many factors affect the wound healing of DFUs, including a long-lasting ulcer, deficiency in either the arteries or veins, the patient’s general health status and medication usage, neuropathy, poor nourishment, and bacterial infection (Jalilian et al., 2020). Among them, infection is the main factor contributing to delayed healing of diabetic foot wounds (Bader, 2008). In addition, ischemia, and hyperglycemia, were also closely associated with the fundamental mechanisms leading to chronic wounds (Syauta et al., 2021). Several physiologic and biochemical abnormalities, including prolonged inflammation, imbalance in extracellular matrix (ECM) synthesis and degradation, poor neovascularization, and impaired macrophage activity, are known to hinder the wound healing process (Clayton and Elasy, 2009). In all these processes, the matrix metalloproteinases (MMPs) seem to be involved.

## Overview of MMPs

**FIGURE 1:**
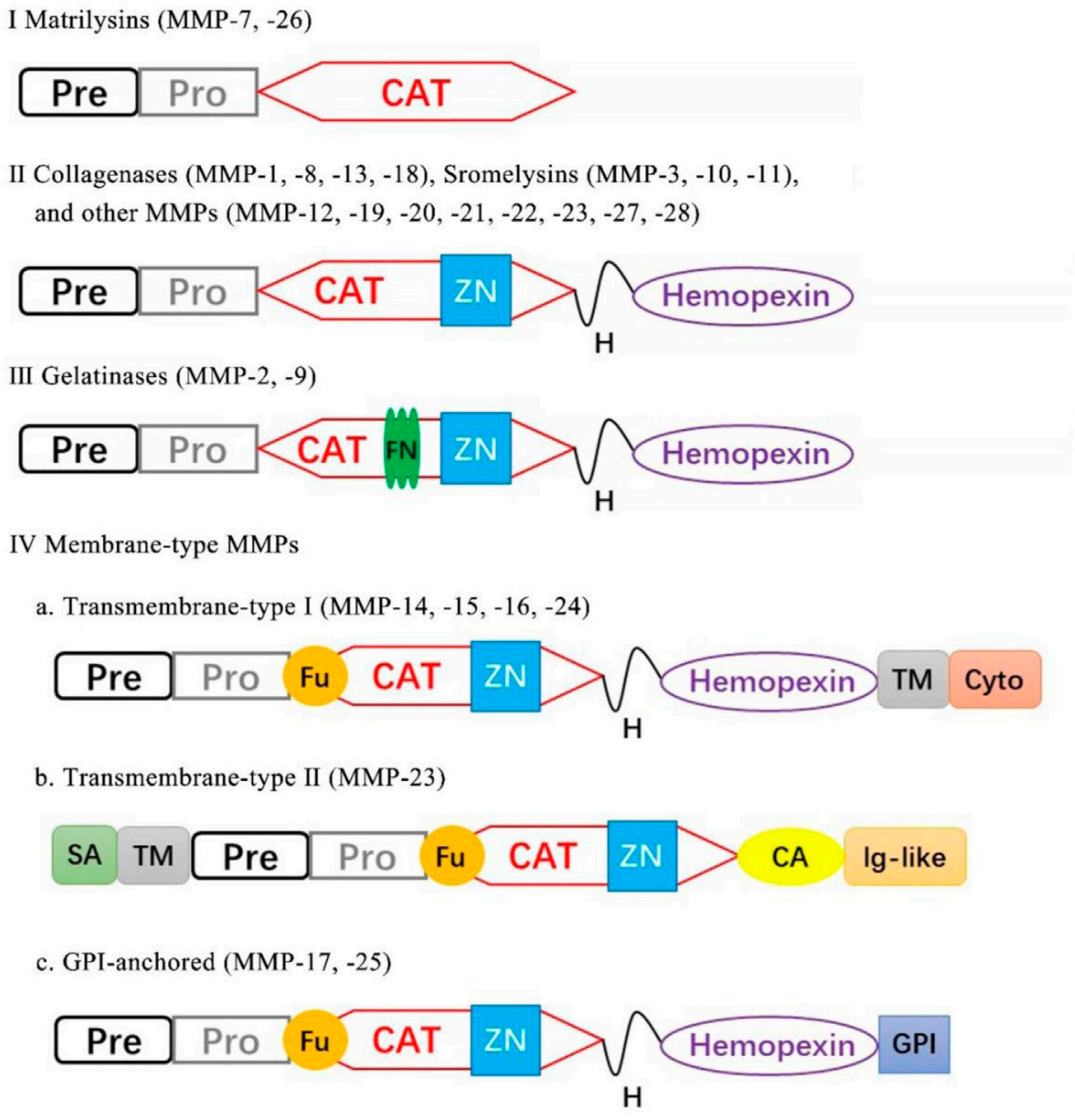
Classification of MMPs according to their domain structure. Pre, signal peptide; Pro, propeptide; CAT, catalytic; ZN, zinc-binding site; Fu, furin cleavage site; TM, transmembrane; Cy, cytoplasmic; SA, type-II signal anchor, H, hinge region; GPI, GPI anchor; FN, fibronectin repeat.

The MMPs belong to a family of zinc-containing proteolytic enzymes that were first discovered in tadpoles with the function of the degradation of ECM (Gross and Lapiere, 1962; Kaur et al., 2020). All MMPs share a structural similarity and several domains in common: 1) an NH_2_-terminal signal sequence (signal peptide) guiding MMPs to the secretory or plasma membrane insertion pathway; 2) a pro-peptide domain occupies the active site, blocking substrate access to the catalytic enzyme. The enzyme is activated by the cleavage of the pro-peptide domain; 3) catalytic domain, a zinc ion binding domain; 4) hinge domain, following the catalytic domain and followed by hemopexin domain; 5) hemopexin-like C-terminal domain, mediating interactions with substrates and granting enzyme selectivity. The hemopexin domain can be found in all the MMPs except MMP-7 (Nagase et al., 2006). According to the substrate specificity and domain organization, MMPs can be categorized as follows: matrilysins, collagenases, gelatinases, stromelysins, membrane-type MMPs, and other MMPs (Zítka et al., 2010) (Figure 1).

MMPs are produced as pro-MMPs, which are inactive zymogens that need protease removal of pro-domain to become active. Active MMPs are mediated by tissue inhibitors of matrix metalloproteinases (TIMPs), including TIMP-1, 2, 3, and 4 that obstruct access to the catalytic site and regulate MMPs activity. An imbalance between TIMPs and MMPs has been linked with the pathogenesis and progression of several diseases (Gill and Parks, 2008; Brew and Nagase, 2010). MMPs participate in cell-cell contacts and cellular interactions with the ECM by modifying the amounts of cytokines, hormones, and ECM components. MMPs indirectly affect cellular activity by modifying membrane receptors, junctional proteins, and diverse physiological activities, such as cell apoptosis and inflammatory processes. In normal skin or acute wounds, the expression of MMPs is maintained at minimal levels, however, their levels are elevated in chronic wound healing involving ECM remodeling(Armstrong and Jude, 2002).

## MMPs in normal wound healing

During normal wound healing, MMPs play an important role. They are involved in each stage of the recovery process: removing the disrupted protein, degrading the temporary ECM, promoting the recruitment of related cells to the wound, restructuring the granulation tissue, and regulating angiogenesis and the expression of growth factors (Kandhwal et al., 2022). Despite their importance in each stage of wound healing, MMPs expression are intricately modulated. During the homeostasis/inflammatory phase, the inflammatory cells are recruited to the injured site through the secretion of chemical messengers from platelets and release MMPs to degrade damaged ECM. This not only aids in the removal of damaged and dead tissue but also ensures the proper interaction of newly produced ECM at the wound edge. During the proliferation phase, MMPs act on the arteriole basement membranes and promote the formation of granulation tissue. MMPs facilitate the migration of endothelial cells, fibroblasts, keratinocytes, and vascular endothelial cells across the ECM to the wound bed (Chen and Parks, 2009). In response to growth factors, endothelial cells of existing blood arteries initiate signaling cascades that culminate in the production of MMPs, thereby enabling endothelial proliferation and migration *via* digestion of tissue matrix (Martins et al., 2013). MMPs-regulated fibroblasts migrate to the wound site and synthesize both collagen and elastin fibers, which are necessary for granulation tissue formation. The cytokines generated by fibroblasts and platelets, which are regulated by MMPs, stimulate angiogenesis, allowing the migration of vascular endothelial cells from blood vessels close to the healing wound thus forming new vessels in the wound bed (Darby et al., 2014). Similarly, migrating keratinocytes release MMPs that alter keratinocyte motility by degrading proteins involved in cell-cell and cell-matrix adhesion, promoting re-epithelialization (Raja et al., 2007). During the remodeling phase, myofibroblasts produce MMPs that aid in the contraction of freshly scar tissue (Darby et al., 2014). Thus, MMPs are important at every stage of wound healing, and it is believed that uncontrolled activity of these proteases is one of the main reasons why wounds do not heal properly.

## MMPs in DFUs

MMPs are crucial in the wound healing process. However, if MMPs are present in high amounts in a wound for an extended period and at the wrong moment, they could slow down wound healing by destroying proteins that are essential for wound recovery. Numerous studies have shown that MMPs are highly expressed in DFUs (Izzo et al., 2014) (Table 1). The elevated MMPs are believed to be responsible for poor wound healing because these MMPs degrade the components of the ECM and impede growth factors, both of which are essential for wound healing.

**TABLE 1:** The expression level of MMPs in DFUs.

Types	Subgroup	Cells source	Substrates	Significant role in DFUs	DFUs *vs*. normal wound	References
Gelatinases	MMP-2	Fibroblasts, Keratinocytes, Endothelial cells, Macrophages	Gelatines; Collagens III, IV, V, VII, X, XI, XIV	upregulated MMP-2 is detrimental to DFU healing	-^a^	Muller et al. (2008)
					↑	Lobmann et al. (2002)
	MMP-9	Keratinocytes, Neutrophils, Macrophages, Endothelial cells	Gelatines; Collagens I, IV, V	upregulated MMP-9 is detrimental to DFU healing	↑	(Lobmann et al., 2002; Muller et al., 2008; López-López et al., 2014; Jindatanmanusan et al., 2018; Nguyen et al., 2018)
Collagenases	MMP-1	Proliferating and migrating keratinocytes, fibroblasts	Collagens I, II, III, V, VII, X, XI; gelatines	upregulated MMP-1 is beneficial to DFU healing	↓	Muller et al. (2008)
					↑	(Lobmann et al., 2002; López-López et al., 2014)
	MMP-8	Neutrophils	Collagens I, II, III, VII, VIII, X; Gelatines	upregulated MMP-8 is beneficial to DFU healing	-^a^	Muller et al. (2008)
					↓	López-López et al. (2014)
					↑	(Lobmann et al., 2002; Nguyen et al., 2018)
	MMP-13	Fibroblasts, Migrating keratinocytes	Collagens I, II, III, IV, IX, X, XIV	unclear	↑	Castruita-De la Rosa et al. (2017)
Stromelysins	MMP-3	Basal proliferating keratinocytes, Fibroblasts	Collagens I, III, IV, V, IX, X; Gelatines	unclear	↓	Castruita-De la Rosa et al. (2017)
	MMP-10	Migrating keratinocyte, Fibroblasts	Collagens I, III, IV, V, IX, X; Gelatines	unclear	↓	López-López et al. (2014)
Membrane-type	MMP-14	Migrating keratinocytes	Collagen I, II, III; Gelatines	unclear	↓	Bassar et al. (2022)
Others	MMP-19	Keratinocyte, Fibroblast, Endothelial cells	Gelatines; Collagen IV	unclear	-	López-López et al. (2014)

^a^ Overexpressed levels without statistical difference.

↑: upregulation; ↓: downregulation; -: no change.

### Gelatinases

Gelatinases A (MMP-2) and B (MMP-9) are the most prominently upregulated enzymes in chronic wounds. Several studies demonstrate higher levels of MMP-2 and MMP-9 in DFUs than those in acute wounds or normal skin (Lobmann et al., 2002). Gelatinases can degrade a broad spectrum of ECM components that in turn regulate cell growth, migration, and angiogenesis. In chronic wounds, MMP-9, which is produced by inflammatory cells, selectively degrade the growth factors and other components that assist the healing process. Vascular endothelial growth factor (VEGF) and dermatopontin are two proteins that promote wound healing; however, they are destroyed at higher rates by upregulated MMP-9, which renders them ineffectual in wound healing (Christoffersson et al., 2012; Krishnaswamy et al., 2014). The existence of elevated MMP-9 levels indicated the continuation of the inflammatory phase and poor wound healing in DFUs. Numerous investigations have verified that MMP-9 is detrimental to the process of wound healing in DFUs, and these investigations all come to the same conclusion. Wound healing is accelerated in diabetes patients with selective MMP-9 inhibition (Gooyit et al., 2014; Peng et al., 2020) or MMP-9 deletion (Gao et al., 2015). This is supported by the confirmation of the target MMP-9 in debridement tissue taken from human patients diagnosed with DFUs, where DFUs were scaled according to Wagner’s classification. MMP-9 showed significantly greater quantities in grade 3–4 ulcers than in grade 1–2 ulcers, which were significantly greater than in control ulcers (Nguyen et al., 2018). On the other hand, MMP-2 is necessary for normal wound healing. Endothelial cells that have been treated with exogenous MMP-2 have undergone dose-dependent morphologic alterations that are compatible with an angiogenic response (Dean et al., 2007; Dean et al., 2007). However, an overactive MMP-2 protein causes an abnormal breakdown of the matrix and limits the production and remodeling of the new matrix, which can lead to a chronic wound (Zhou et al., 2021). MMP-2 cleaves laminin-332 to stimulate the migration of keratinocytes, and it also releases an epidermal growth factor (EGF)-like fragment of the γ2-subunit short arm. This fragment binds to the EGF receptor and initiates cell movement, and it has the potential to obliterate re-epithelialization when its levels are altered(Schenk et al., 2003).

### Collagenases

MMP-1,8 and 13 can cleave the triple helix of fibrillar collagen, making them the only enzymes in mammals with this ability. High collagenase activity and decreased levels of TIMP-1 were observed in the wounds of DFUs (Hennessey et al., 1990). MMP-1 is primarily accountable for collagenase-related wound healing for its ability to complete the proliferative phase. MMP-1 is responsible for cleaving type I collagen, which is necessary for regulating keratinocyte migration to the injured site and thus re-epidermization (Sudbeck et al., 1997). In DFUs, the MMP-1 concentrations were considerably 65 times greater than in acute lesions from non-diabetic patients (Lobmann et al., 2002). Upregulated MMP-1 is beneficial to the wound healing process and the ratio of MMP-1 to TIMP-1 was used to predict wound healing in DFUs (Muller et al., 2008). Overexpressed MMP-8, which is derived from neutrophils plays a direct role in the etiology of chronic wounds. By breaking down fibronectin, α1-antiproteinase, α2-macroglobulin, growth factors, and products of fibroblast synthesis, MMP-8 plays an important role in the pathophysiology of the wound. Although high levels of MMP-8 were reported in the wounds of DFUs (Muller et al., 2008), a conflicting conclusion was reached about the function of MMP-8 in the wound healing process. Danielsen et al. have evaluated the effect of highly expressed MMP-8 on chronic wound healing and the findings show that overexpression of MMP-8 can degrade collagen and result in impaired wound healing (Danielsen et al., 2011). A recent study, on the other hand, found that topical active recombinant MMP-8 in diabetic wounds boosted the wound healing rates because it led to complete re-epithelialization, less inflammation, and more blood vessel growth (Gao et al., 2015). A diabetic wound that was treated with a selective MMP-8 inhibitor took significantly longer to heal, had significantly less re-epithelialization, and displayed a high level of apoptosis (Gooyit et al., 2014). Confirmation of the beneficial effects that MMP-8 plays in DFUs, in which mice lacking MMP-8 heal their wounds more slowly and exhibit higher levels of inflammatory responses. All the findings point to MMP-8 being an essential component in the healing process of diabetic wounds. A high level of expression of MMP-13 (collagenase-3) is observed in chronic wound beds but not in normally healing wounds (Vaalamo et al., 1997). MMP-13 has been demonstrated to improve the remodeling of dermal fibroblasts’ 3D collagen matrix, cell morphology, and cell survival. It demonstrates that MMP-13 plays particular functions in the production of granulation tissue as well as in the modification of the ECM (Toriseva et al., 2012). In non-healing wounds, MMP-13 exhibits distinct spatial expression patterns (Vaalamo et al., 1997). In the deeper layers of the chronic ulcer bed, fibroblasts showed high levels of MMP-13 expression, although the epidermis showed no signs of it. MMP-13 is not engaged in the normal process of wound healing; nevertheless, it plays a significant role in the remodeling of dermal stroma in chronic wounds, which may impair the wound healing. In general, the findings of this more recent research point to the fact that collagenases are essential for the ECM remodeling that takes place throughout the process of wound healing.

### Stromelysins

Due to their extensive substrate specificity, stromelysins play a variety of roles in ECM degradation. Stromelysin-1 (MMP-3) and stromelysin-2 (MMP-10) regulate collagenolytic activity and wound remodeling. MMP-3 is secreted by proliferating keratinocytes at the distal portion of the wound, while co-localization of MMP-10 with MMP-1 was identified near the leading edge of the wound (Caley et al., 2015). According to research conducted on the function of MMP-3 in wound healing, a lack of MMP-3 causes disrupted dermal wound healing, which results in an impaired ability of fibroblasts to contract the wound *in vivo*. The findings indicate that MMP-3 plays an important role in the process of wound healing. The application of MMP-3 topically resulted in dramatically enhanced angiogenesis and reparative dentin production. Inhibition of MMP-3 activity results in untoward wound healing (Zheng et al., 2009). Importantly, MMP-3 is a physiological activator of MMP-9, therefore increased MMP-9 activity may worsen the inflammatory phase of chronic wound healing. (Ogata et al., 1992). In contrast, animals with aberrant MMP-10 expression have a disorderly migrating epithelium, breakdown of ECM, abnormal cell-cell interactions of the migrating keratinocytes, and a higher rate of cell death in the keratinocytes near the edge of the wound (Krampert et al., 2004).

### Membrane-type MMPs

Members of this class of MMPs have distinct structural characteristics, but they are not secreted into the ECM, which distinguishes them from all other MMPs. Located on plasma membranes, membrane-type MMPs play an important role in the activation or localization of other MMPs (Hernandez-Barrantes et al., 2000). MMP-14 activity is crucial for physiological and pathological processes. Downregulated MMP-14 expression not only controls the activation of MMP-2 and MMP-13 but also prevents the eventual direct degradation of other ECM components (Pincus et al., 2010). In addition, MMP-15 and MMP-16 are both capable of binding to the gelatinases, but not as strongly as MMP-14.

### Other MMPs

Other MMPs such as MMP-12 and MMP-7 have a role in chronic wound pathogenesis. MMP-7 is responsible for stimulating the process of re-epithelialization (Letra et al., 2013). MMP-12 is induced by Agrin and mediates collective migration due to enhanced local inflammation (Aristorena et al., 2019; Chakraborty et al., 2021). Although chronic cutaneous wounds in humans have not been extensively studied, these MMPs play a key part in wound healing because of their ability to influence variety of cellular processes and activate other MMPs.

**FIGURE 2:**
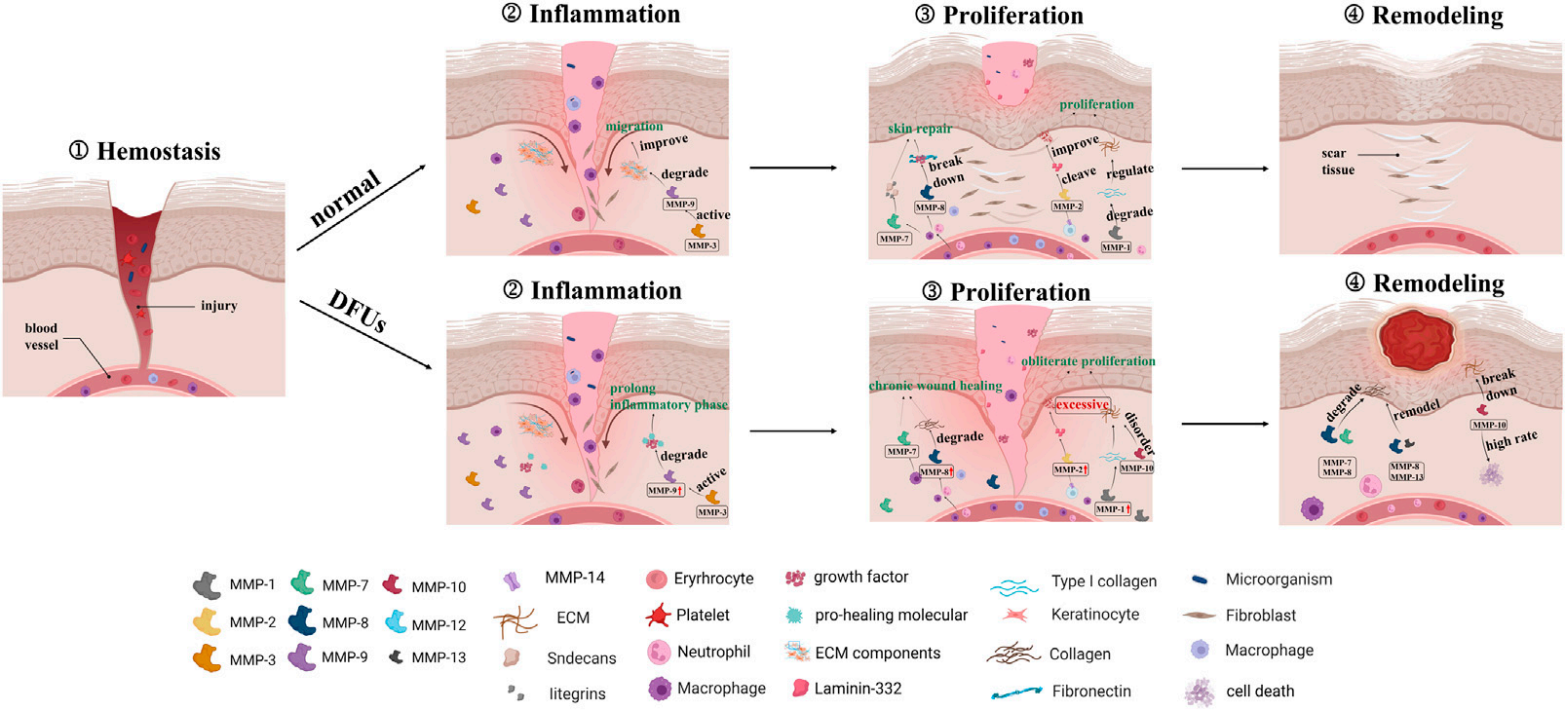
The graphical depiction of normal and chronic wound healing with dysregulated MMPs.

The expression of MMPs during a normal wound are accompanied by a release of growth factors from inflammatory cells, resulting in limited and transient inflammatory factor stimulation. The immune cells secrete MMPs to remove local bacteria and degrade ECM from the wound as part of an orderly inflammatory response. However, in DFUs, recurrent tissue injury prolongs and exacerbates this inflammatory state, resulting in a cascading effect between inflammatory cytokines and macrophages in the wound. Increased inflammatory cells secrete large amounts of inflammatory cytokines, which directly stimulate excessive MMP expression. In addition, high levels of TNF-α and IL-1 not only directly induce the secretion of MMPs, but also suppress the formation of TIMPs. The upregulated MMPs degrade the necessary wound-healing components, resulting in the development of chronic wound healing. The “hidden damage” to the skin that exists before wound formation may further exacerbate the inflammatory response and slow wound healing after wound formation. The overview of different MMPs and their dysregulation in normal and DFUs wound is schematically illustrated in Figure 2.

## Targeting MMPs inhibition in DFUs

To date, most of results indicated that overexpression of MMPs associated with the chronic healing process in DFUs. MMP inhibitors have thus been developed for the treatment of DFUs. Most of the research for treating DFUs falls into the following categories:

### Small molecule inhibitors

Although MMPs exist in multiple forms (pro-MMPs, active MMPs, and TIMP-MMPs) *in vivo*, only the activated MMPs exert pharmacological activity. To identify and quantify the active MMPs in DFUs, an affinity resin was developed to extract the active MMPs from DFUs. Using this method, they have identified active MMP-8 and MMP-9 in DFUs. Next, they developed a specific MMP-9 inhibitor, ND-336, which has *K*_i_ values of 150 nM for MMP-9 and 7700 nM for MMP-8. This indicates that 50 times more selective for MMP-9 than it is for MMP-8. In an *in vivo* animal study, the topical application of ND-336 sped up the recovery time of diabetic wounds. *In situ* zymography studies have shown that ND-336 had a strong inhibitory effect on MMP-9 activity while not affecting MMP-8 activity (Gao et al., 2015). As racemic ND-336 showed efficacy in diabetic wounds, they also isolated the (*R*) and (*S*)-enantiomers. (*R*)-ND-336 is a more potent MMP-9 inhibitor than its counterpart, (*S*)-ND-336. *In vivo*, wounds treated with (*R*)-ND-336 showed a full re-epithelialization and complete MMP-9 inhibition, whereas partial inhibition was observed after (*S*)-ND-336 treatment. Moreover, studies have shown that (*R*)-ND-336 is more efficacious when compared to becaplermin, which is the sole drug currently licensed by the FDA for the management of DFUs (Nguyen et al., 2018; Jones et al., 2019; Peng et al., 2020). All the results show that (*R*)-ND-336 has great potential to become a candidate drug for the treatment of DFUs.

### Antibody inhibitors

Developing antibody drugs as MMP inhibitors can significantly enhance the specificity and targeting of MMPs. Numerous inhibitory antibodies against MMPs have been developed, such as andecaliximab (GS-5745), a specific dual inhibitory antibody against MMP-9 (Jones et al., 2019; Kaur et al., 2020). It inhibits MMP-9 activity in two ways: directly, by interfering with the active site of MMP-9, and indirectly, by interfering with the MMP-3 zymogen, which is involved in the activation of MMP-9 (Sela-Passwell et al., 2011; Appleby et al., 2017). Recent research has reported that GS-5745 was well-tolerated in phase I clinical trials (Bendell et al., 2015; Shah et al., 2018). Clinical research has also shown that two additional antibodies, SDS3 (Sela-Passwell et al., 2011) and REGA-3G12, specifically block MMP-9 activity (Paemen et al., 1995; Hu et al., 2004). But all these three antibodies are currently used only for anti-tumor research, not wound healing. However, these gradual in-depth studies on MMP-9 inhibitors show that these targeted inhibitors have great research value and potential in DFUs treatment (Hariono et al., 2018).

### RNA interference

The use of RNA interference (RNAi) to suppress the production of a specific protein has shown great promise as a method for treating a wide range of diseases. Therefore, therapeutic approaches that interfere with the expression of MMPs at the genetic level have significant potential in the treatment of diabetic wounds (Wang et al., 2010). Diabetes wounds treated by MMP-9-siRNA exhibit reduced MMP-9 levels. Wound healing is dramatically enhanced by its ability to decrease MMP-9 gene expression after being absorbed by fibroblasts (Li et al., 2014). In subsequent experiments, however, it was discovered that siRNA caused liver and kidney damage and a short duration of pharmacological effect (Li et al., 2017). Despite showing great promise in the treatment of DFUs, delivering small interfering RNA (siRNA) safely and effectively remains a challenge (Srinivasachari et al., 2008; Xu et al., 2009; Wang et al., 2010). Recently an effective and safe siRNA delivery system, named *β*-CD-(D3)7, was explored to deliver siRNA effectively in diabetic wounds. When administered, *β*-CD-(D3)7/MMP-9 siRNA reduced the expression of MMP-9, which in turn accelerated wound healing without causing tissue accumulation or toxicity (Li et al., 2017). Nevertheless, repeated dosing was needed for long-term silence of MMP-9, which may be harmful to the healing process. Furthermore, *β*-CD-(D3)7/siMMP-9 NPs can enter the bloodstream and accumulate in the liver, where it has the potential to cause liver toxicity. Herein, a hybrid hydrogel dressing was designed to aid in the localization of siMMP-9 delivery while also extending its duration (Lan et al., 2021). Temperature-sensitive controlled release of siMMP-9 entrapped in wound bed allowed targeted and sustained administration *in vivo*, which silenced MMP-9 expression and dramatically enhanced diabetic wound healing without systemic toxicity.

### MMP modulating dressings

It is normal practice to use wound dressings to provide a moist wound environment, protect from bacterial infection, remove exudate secretion, and promote wound healing in DFUs (Hilton et al., 2004; Vowden and Vowden, 2017). A dressing composition containing an MMP inhibitor has been developed. When the dressing is applied to a wound, the MMP inhibitor contacts the wound fluid to selectively inhibit one or more MMPs in the wound bed without affecting the MMP levels for normal wound healing (Rayment et al., 2010). A patented wound dressing containing MMP in a barrier layer was designed for DFUs. When in contact with a wound, the layer breaks down, releasing the therapeutic compounds into the wound (Varma et al., 2006). Although MMP inhibitors have been applied to these wound dressings, it has been observed that these inhibitors are generally nonspecific (Rayment et al., 2008). The pharmacological effect of non-selective MMP inhibition is not good as that of specific MMP inhibition (Agren et al., 2001). So related studies on specific MMP inhibitors are still worthy of further research and attention.

## Conclusion and perspective

DFUs are the common cause of non-traumatic amputations in developed countries. Until now, there is a lacking effective medical treatment for this chronic wound. Understanding the etiology of these chronic wounds will allow for more effective treatment. Recently, numerous studies have shown that MMPs are overexpressed in chronic wounds, and inhibiting MMPs (especially MMP-9) activity as a potential treatment for DFUs has also gained a lot of research interest. Even though there has been a lot of progress and some promising candidate drugs have emerged, there are still some issues. First, due to their widespread involvement in normal wound healing and physiological process, it is unclear if altering MMP expression for DFUs treatment will also result in unintended effects. Second, for the structural and morphological similarity of MMP family members, it is challenging to target specific MMPs, particularly when they belong to the same category (Overall and Kleifeld, 2006; Amar et al., 2017). Although there are a lot of related types of research on broad-spectrum MMP inhibitors (MMPIs) (Gooyit et al., 2014; Levin et al., 2017), more selective and specific MMP inhibitors are still needed. Third, the MMP expression undergoes a dynamic change and the amounts of MMPs at each stage of diabetic wound healing are completely different. Can it be speculated that the therapeutic effect of MMP inhibitors will also be greatly different? Whether MMP inhibitors should be strictly stratified before deciding whether to use them in clinical application. Therefore, it is still required to have a comprehensive understanding of the roles that MMPs play in DFUs to find new MMP inhibitors and alternative targeted MMP therapies.
